# Harnessing Extracellular Matrix Biology for Tumor Drug Delivery

**DOI:** 10.3390/jpm11020088

**Published:** 2021-01-31

**Authors:** Nithya Subrahmanyam, Hamidreza Ghandehari

**Affiliations:** 1 Department of Pharmaceutics and Pharmaceutical Chemistry, University of Utah, Salt Lake City, UT 84112, USA; nithya.subrahmanyam@utah.edu; 2 Utah Center for Nanomedicine, University of Utah, Salt Lake City, UT 84112, USA; 3 Department of Biomedical Engineering, University of Utah, Salt Lake City, UT 84112, USA

**Keywords:** extracellular matrix, drug delivery, tumor, cancer, targeting

## Abstract

The extracellular matrix (ECM) plays an active role in cell life through a tightly controlled reciprocal relationship maintained by several fibrous proteins, enzymes, receptors, and other components. It is also highly involved in cancer progression. Because of its role in cancer etiology, the ECM holds opportunities for cancer therapy on several fronts. There are targets in the tumor-associated ECM at the level of signaling molecules, enzyme expression, protein structure, receptor interactions, and others. In particular, the ECM is implicated in invasiveness of tumors through its signaling interactions with cells. By capitalizing on the biology of the tumor microenvironment and the opportunities it presents for intervention, the ECM has been investigated as a therapeutic target, to facilitate drug delivery, and as a prognostic or diagnostic marker for tumor progression and therapeutic intervention. This review summarizes the tumor ECM biology as it relates to drug delivery with emphasis on design parameters targeting the ECM.

## 1. Introduction

Targeted drug delivery capitalizes on biological aspects of the tumor ECM, and can thus be informed by an understanding of the intricate dynamics that affect the tumor microenvironment. Many drug carriers are modified to target specific upregulated biomarkers, proteins, receptors, and other epitopes within the tumor ECM in order to increase localization by capitalizing on a biological change in the tumor microenvironment compared to healthy tissue. Here we summarize both drug delivery and cancer biology literature to understand the local dynamics that influence drug delivery.

The ECM, the complex non-cellular environment, is essential to cell processes [1]. It has a reciprocal relationship with cells, providing signaling cues that influence nearly all aspects of cell life [2]. Once thought to be merely a structural support, the ECM is now well-recognized to have a homeostatic relationship with cells maintained through biochemical and mechanotransducive interactions [3]. Homeostasis is maintained through a tightly structured enzymatic processing of ECM components. In cancer, this homeostasis is disrupted in favor of promoting excessive growth of cells and an invasive phenotype [4]. The ECM presents opportunities to target, treat, and modulate the stroma, as well as epitope targets and biological mechanisms that can be harnessed for therapeutic intervention [5]. For example, the contribution of various ECM enzymes to tumor growth and invasion has led to development of therapeutics based on enzyme inhibition strategies. Additionally, the biochemical and morphological changes in the ECM can be interpreted as diagnostic and prognostic markers [6,7].

In this review, we take a comprehensive look at the many ways that the ECM can be used as a tool for cancer treatment. To this end, we first discuss the complex biology of the ECM, its function and composition, and the changes it undergoes in cancer. We then examine efforts to employ it as a therapeutic target and as a diagnostic and prognostic marker, as well as strategies to prime the ECM to improve drug delivery through small molecule approaches and mechanical or enzymatic strategies. Finally, we examine efforts to further improve delivery through the use of drug carriers by ECM targeting or modulation.

## 2. Extracellular Matrix: Structure, Function, and Involvement in Cancer Etiology

In this section, we provide background for ECM-based therapeutic strategies by presenting an overview of the biology of the healthy ECM, its components, and its structure. We then discuss the changes induced by cancer in the ECM, which present epitopes for targeting and pathways to hijack for therapeutic intervention. Finally, we highlight some of the differences between healthy and tumor ECM. These ideas together provide a basis for a biological platform that can be leveraged for ECM-based strategies.

### 2.1. Function and Role of the ECM

The ECM firstly serves as a physical scaffold that helps to maintain the structure of organs [8]. It delineates tissue boundaries, preventing unnecessary cell migration and abnormal proliferation, and provides elasticity for organs [8,9]. Providing elasticity is particularly important during development and morphogenesis [10].

Next, the extracellular matrix serves as an adhesive substrate to facilitate cell migration. The mechanisms of cell adhesion are carried out through a set of molecules and receptors collectively known as the adhesome [11]. The adhesion sites help connect cells with their neighboring cells [12]. Additionally, with respect to the ECM, these adhesion molecules are important for environmental sensing, including for both chemical and physical properties [12]. Adhesion interactions occur through both integrin and non-integrin receptors. Integrins are transmembrane proteins composed of an α subunit and a β subunit. They interact with molecules in the ECM. There are 18 different α chain subunits and 8 different β chain subunits, which give many possible heterodimeric integrins [12]. The adhesome molecules are substrates for attachment, as well as mediators involved in the growth and remodeling of the ECM [11,13,14]. Integrins are stimulated by both mechanical (detecting stiffness of the ECM) and biochemical cues which instigate a conformational change, leading to downstream biochemical responses that modulate cell behavior [15,16,17]. Specific integrins on cells sense corresponding proteins and epitopes from the ECM [16]. Several matricellular proteins (including fibronectin [18], collagen, and others) are recognized by integrins and participate in the ECM–cell communication. These proteins are then further connected by a web of interactions with cells and other ECM components. Integrins and adhesion dynamics offer many opportunities for drug development and targeting [19,20,21].

Related to this environmental sensing that facilitates cell migration, an interesting and complex role that the ECM plays is in ECM–cell signaling, which is facilitated through multiple mechanisms. Fibrous proteins and glycosaminoglycans in the ECM bind growth factors and serve as a repository of embedded molecules which then get released in a regulated fashion through enzymatic processing [22]. These molecules are presented to the cell surface and in turn activate cellular pathways, in addition to direct ECM molecular interactions with cell surface receptors, facilitating intracellular signaling [22,23]. Examples of the ECM molecules that serve as reservoirs include heparan sulfate and heparan, collagen, and others. Examples of bound growth factors include vascular endothelial growth factor (VEGF), fibroblast growth factors (FGF), and others [24]. Remodeling of ECM molecules is facilitated predominantly by matrix metalloproteinases (MMPs) [25]. In some cases, the ECM molecule instead serves as a cofactor in the binding of growth factors to their cognate receptors [26].

In addition to the biochemical dynamics of these components, biomechanics also plays an important role in the ECM, both in terms of maintaining homeostasis as well as ECM–cell signaling [3]. The ECM communicates with cells through mechanotransduction [3]. In this process, the fibrous structure of the ECM exerts a mechanical pressure on the cell surface, where the tensile strength conveys signals [3]. The density and alignment of the fibrous structures both play an important role in this process [6].

The mechanical and dynamic homeostasis of the ECM is critical [3]. Homeostasis is maintained through effector cells and sensing. In the case of fibrous collagen, fibroblasts are important as effector cells in this process, as they secrete both collagen and proteases such as matrix metalloproteinases that can break down collagen in response to cues [3]. This breakdown maintains homeostasis within the ECM and with respect to cells and cellular function [27]. Disruptions to this homeostasis matter because they are both symptomatic and causative of pathological conditions, and often promote disease progression.

### 2.2. Composition of the ECM

The composition of the ECM varies depending on the type of ECM and the location. There are approximately 300 proteins in the mammalian ECM [9]. ECM material is secreted by fibroblasts, and it is in general made up of proteins (such as collagen, elastin, and fibronectin), glycosaminoglycans (largely hyaluronic acid), and proteoglycans (heparan sulfate and others). It interacts with cells and organs and maintains a tensile and compressive force [2]. It is also comprised of growth factors and signaling molecules, and it houses various immune and other cells, such as fibroblasts. The most abundant constituent is collagen, with the interstitium containing primarily collagen type I [28]. Fibroblasts secrete collagen and help organize its structure [29]. Elastin is secreted as tropoelastin, assembled into fibers, and tightly associated with the collagen, providing elasticity [2]. Fibronectin is involved in organizing the ECM, mediating cell functions and interaction, and its unfolding is mediated by mechanical forces [2]. Proteoglycans, which are made up of proteins covalently attached to glycosaminoglycans, are involved in interactions with growth factors and other signaling molecules, playing a role in the organization of the ECM structure [1]. Hyaluronan, a glycosaminoglycan, is involved in mediating several functions through binding to cell receptors [1]. Additionally there is a milieu of enzymes, which continually remodel the ECM and maintain homeostasis. Some examples of these include MMPs, a disintegrin and metalloproteases (ADAMs), a disintegrin and metalloproteases thrombospondin motifs (ADAMTs), heparanase, and many others [1]. The interstitium is connected to a basement membrane which separates it from blood vessels [30]. The basement membrane is predominantly composed of collagen type IV and laminin [30]. All of these molecules have receptors with which they interact in order to facilitate communication between cells and the ECM. Specific components of the ECM that are important targets for delivery are described in more detail later.

### 2.3. Pathological ECM

Hanahan and Weinberg famously described the hallmarks of cancer [31], and Pickup et al. extended this concept to how the extracellular matrix contributes to each of these hallmarks [32]. Cues from the ECM play a role in influencing each of these hallmarks, attesting to the integrative nature of cancer with its environment, and these are in fact essential to the development of malignancies [32]. In cancer, the ECM processes are dysregulated to collectively promote tumor growth and metastasis.

#### 2.3.1. Enzyme Upregulation

**Figure 1:**
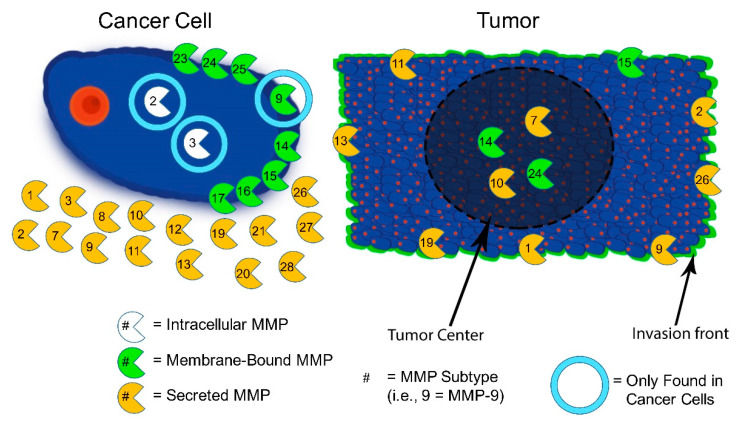
Schematic depicting the distribution of matrix metalloproteinase (MMP) subtypes within the tumor microenvironment. The cancer cell on the left, depicts intracellular MMPs (shown to the right of the nucleus, represented in red), membrane-bound MMPs, and secreted MMPs. Additionally, the light blue circle indicates those MMPs that are found in that particular location only in cancer cells (e.g., MMP-2 is found intracellularly in cancer cells, but not in healthy cells). The tumor microenvironment on the right (depicted with a background of cancer cells for schematic representation) shows the distribution of MMPs within the tumor microenvironment, indicating those present near the tumor interior versus near the periphery. Image adapted with permissions from Isaacson et al., 2017 [25].

Numerous enzymes (such as MMPs and cathepsins) are upregulated in the tumor ECM, which promote degradation and weakening of the basement membrane (although the relationship with these enzymes is more complex, with some enzymes showing both pro- and anti- tumor effects [33,34]). A summary of ECM enzymes is given in Table 1. Additionally, it is important to consider the distribution of enzymes and molecules within the tumor microenvironment, particularly in the context of targeting. Figure 1, adapted from Isaacson et al. [25], summarizes the distribution of MMP enzyme subtypes within a tumor microenvironment. Enzyme inhibition is a common disease therapy, and Table 2 gives examples of enzyme inhibitors with corresponding enzymes. The list is by no means exhaustive, but highlights some of the more studied examples of small molecules, drugs, and others and their associated matrix components. Increased enzymatic remodeling of various constituents primes the microenvironment for cancer through breakdown of the matrix to allow cell migration, weakening of the basement membrane to permit escape, increased growth, and increased presentation and release of pro-tumor cues [4]. Additionally, enzymatic remodeling leads to exposing cryptic domains within ECM protein structures, which enables binding of signaling factors that promote growth and angiogenesis [30]. This includes largely MMPs as well as cathepsins. Cathepsin K, for example, is responsible for bone resorption [35], and it is upregulated in breast cancer tumors that metastasize to the bone. Cathepsin B is involved in weakening of the basement membrane, promoting metastasis [36]. These also vary by tumor type and subtype. The differences attest to the heterogeneity of tumor stroma profiles. Another example, tissue-type plasminogen activator (tPA), is known to be secreted by cancer cells, and it is associated with several proteolytic channels, including fibrin degradation, and coagulation and complement systems [1]. This can lead to fibrin clots, leading to a restriction of tumor vascular permeability. Fibrin blood clots are degraded by plasmin, which is produced through the digestion of plasminogen via tPA [1]. These various enzymatic changes and processes are essential to cancer progression and metastasis.

#### 2.3.2. Weakening of Basement Membrane

The basement membrane is thinner in invasive tumors due to remodeling, and it contains significantly lower levels of laminin (the key structural component of the basement membrane along with collagen type IV) [16]. The basement membrane serves as a barrier between the epithelial cells and the interstitium, and weakening it promotes escape [46]. Invasion is triggered by interactions (degradation) between membrane-bound matrix metalloproteinases (MT-MMPs) and basement membrane. This physical interaction triggers a cross-talk between several factors which then facilitate the process [46].

#### 2.3.3. Increased ECM Deposition and Stiffness

Next, there is an increased deposition of ECM material. One example is an increased deposition of collagen (desmoplasia) [7]. There is also increased alignment of collagen and increased cross-linking. Cross-linking is due to lysyl oxidase activity [47]. This leads to a higher level of mechanical rigidity and stress [2]. These changes are correlated with and further promote tumor growth and invasion, by mechanotransducive signaling (through density, cross-linked stiffness, and fiber alignment) on the cells [3]. Collagen is an integral component of the ECM landscape and it plays an important role in the biophysical communication between ECM and cells, and it has been shown to be highly involved in metastasis [17].

#### 2.3.4. Causative Factors and Pathways

Much of the tumor ECM is synthesized and secreted by cancer-associated fibroblasts (CAFs) [48]. Additionally, stromal cells produce enzymes that digest the basement membrane, which contributes to invasion [48]. Signaling molecules and growth factors play a role as well. For example, TGF-β is particularly important in this cascade in activating fibroblasts [48]. Other important pathways include FAK, ERK, and FGF [49]. Similarly, proteoglycan changes also influence these processes [49]. There are higher levels of proteoglycans, such as chondroitin sulfate and heparan sulfate, as well as variations in enzyme expression. Ultimately, when considering the ECM in tumor progression, it is necessary to consider the effect of the cells on the stroma and the effect of the stroma on the cells [48].

### 2.4. In Summary

The involvement of the matrix in causing invasive phenotype is being analyzed at the most fundamental level: the interactions between a cell’s local environment and physical cues, and the physical forces translated to biochemical signals, attesting to its complexity and importance [50]. Different cancers (such as breast cancer and prostate cancer for instance) display different stroma profiles [46]. The literature sometimes shows varying conclusions about the stroma profiles in different studies, suggesting heterogeneity. A comprehensive and detailed picture of stroma profiles is important because it is common to make conclusions and generalize about upregulated enzymes in the context of drug delivery, while specifics about the site of the enzyme within the microenvironment and the quantity of the enzyme are not always specified.

Ultimately these changes in the tumor ECM are important because they present new targets and avenues in the treatment of cancer. By targeting the mechanisms and components that play a supportive and causative role in tumor progression and metastasis, we can address the complex integration of cancer with its environment, modulating the ECM in conjunction with cytotoxic approaches. Furthermore, understanding the local architecture and interactions is not only important in the context of ECM-based strategies, but in any tumoral drug delivery, as these local dynamics influence migration of drugs and carriers to cells.

## 3. Harnessing ECM Biology

With an understanding of matrix biology, research efforts are underway to exploit this information for cancer treatment. This includes enhancing drug delivery to tumor cells by priming the ECM, as well as designing drugs that act on ECM targets and mechanisms. Additionally, upregulated matrix components are used as prognostic and diagnostic markers. The strategies which focus on either small molecule, enzyme-based, or other modalities are discussed in this section. The section following it discusses drug delivery approaches to further improve targeting.

### 3.1. Priming the ECM to Enhance Drug Delivery

Because the matrix poses a barrier to the migration of drugs and is often cited as the reason for the failure of many treatments, there have been approaches to enhance drug delivery through breaking down the ECM using enzymes such as hyaluronidase. Clinical trials have explored the use of PEGylated hyaluronidase (PEGPH20) in combination with the chemotherapeutic eribulin mesylate, as a means to break down the hyaluronic acid barrier in the ECM [65,66]. Hyaluronidase digests the ECM allowing facile diffusion of the drug molecules to the target. These are particularly relevant in cancers such as pancreatic cancer which have an especially dense stromal matrix. Similarly, it has been shown that administering collagenase improves penetration [67].

In addition to enzyme-based strategies, mechanical strategies are also employed to facilitate drug delivery. An example of priming the matrix to enhance drug delivery is in the use of pulsed high intensity focused ultrasound (HIFU) to alter the collagen structure of the ECM to allow for better penetration of chemotherapeutic drugs [68]. HIFU can be used to induce localized hyperthermia [69], and it can also disrupt collagen structure [68]. Mice inoculated with A549 tumors and administered pulsed-HIFU exhibited increased penetration of chitosan nanoparticles, due to disruption of the ECM. The porosity of the ECM was shown to be increased with higher intensity of the administered ultrasound.

Another strategy is the direct modulation of the cancer-associated fibroblasts (which deposit ECM material) to reduce ECM deposition. An example is the use of all-*trans* retinoic acid (ATRA), which induces quiescence in pancreatic stellate cells (PSCs) of pancreatic ductal adenocarcinoma (PDAC) [70]. Inducing quiescence in PSCs restores homeostasis in the PDAC ECM, resulting in less ECM deposition. This allows for better penetration and delivery of drugs. Lysyl oxidase inhibition has also been shown to improve delivery, as lysyl oxidase, discussed earlier, is responsible for cross-linking the ECM proteins and thus increasing the tumor stiffness [71]. Inhibiting lysyl oxidase showed the ability to potentiate the delivery of other treatments [71].

### 3.2. ECM Molecules as a Therapeutic Target

The ECM can be modulated using small molecules as a therapeutic strategy. Many of these rely on either the signaling pathways between the ECM and cells or on enzyme inhibition. Some examples of ECM therapeutic targets include thrombospondins [72], osteopontins [72], periostins [72], tenascins [72], matrix metalloproteinases [73,74], and cathepsins [75]. Inhibitors are often based on epitopes and binding motifs that are inspired by endogenous substrates.

One example is cathepsin K (Cat K), a cysteine protease responsible for osteoclast-mediated bone resorption. Cat K degrades collagen type I by cleaving the triple helices at different sites, and it has been implicated in cancers with skeletal (bone) metastases [60]. It is found to be upregulated in several tumor types (including bone, lung, prostate, and breast cancer), and specifically more expressed in cancers that are more invasive and metastasize to the bone [75,76,77,78]. Modulation of Cat K activity using Cat K inhibitors influences osteolysis [60]. Though cathepsin K inhibitors have predominantly found their home in the treatment of osteoporosis, they have also gone through clinical trials to treat metastatic bone disease [75]. They have been shown to reduce osteolytic lesions (indicative of metastasis) in breast cancer [60,75].

Another example is matrix metalloproteinases (MMPs), a zinc-dependent family of proteinases which is most implicated in matrix degradation, which plays an important role in invasion and metastasis in the context of cancer [79]. These are located either bound to cell surfaces, within the interstitium, or near the periphery [25]. MMP inhibitors have gone through clinical trials, albeit with limited success towards cancer treatment [80]. Some notable examples include batimastat [51], marimastat [79], and several others. Many of these are broad spectrum inhibitors which coordinate with sites on the enzyme (mimicking the enzyme’s endogenous peptide substrate) and act through chelation of the essential zinc, most classically through a hydroxamic acid moiety [79]. The first generation of MMP inhibitors largely failed due to lack of specificity and off-target toxicities [55].

Proteins, glycoproteins, and proteoglycans additionally are therapeutic targets. Osteopontin (OPN) is a matricellular phosphoglycoprotein that binds to integrins to facilitate ECM–cell communication. Its upregulation promotes tumor progression through several interactions and cascades [81,82]. Osteopontin interacts with several integrins (both with and without its RGD motif), as well as with CD44 receptors [82]. It plays a structural role in the ECM, and it binds to collagen and other proteins [83]. Osteopontin has been shown to be upregulated in several types of cancer, and it is thought to be involved in tumor proliferation, metastasis, induction of angiogenesis, and potentially chemoresistance [82,84]. Osteopontin inhibitors are investigated for cancer treatment, at the level of gene delivery as well as small molecule inhibitors [72,81,82,84].

Thrombospondin is another example that has been explored as a target. Thrombospondins are a family of proteins that regulate cell phenotype and ECM structure [85]. They are suggested to have both supportive and suppressive roles in metastasis, complicated by the fact that they have interactions with numerous other proteins [85]. Thrombospondin-1 regulates angiogenesis, and inhibiting it leads to enhanced vasculature formation, which creates a leakier vasculature in order to enhance extravasation and delivery. However, it is also an inhibitor of tumor growth, making the interaction more complicated [86]. Additionally, thrombospondins are thought to activate TGF-β in some tumor types but not others, further complicating its influence on tumor progression [86]. Inhibitors of thrombospondin have had more progress associated with inhibiting fibrosis [87].

Heparan sulfate proteoglycans have also been explored as a target in breast cancer therapy, because they participate in signaling pathways involved in tumor progression [88]. Heparan sulfate binds growth factors that are involved in signaling angiogenesis, metastasis, and tumor proliferation [89]. However, heparan sulfate also contains growth factors that are cancer inhibitory, so its role is complex [89]. Furthermore, heparan itself is also explored as a therapeutic molecule [89].

In summary, the complexity of the ECM interactions offers many opportunities that are very complicated since many proteins have both pro- and anti- tumor properties, emphasizing the importance of homeostasis.

### 3.3. ECM as a Prognostic and Diagnostic Biomarker

The ECM has also been utilized as a biomarker for its tight correlation with cancer stage. One well-established example is collagen [90]. Collagen radial alignment has been recognized as a prognostic signature for tumor advancement [6]. This has been termed TACS (tumor associated collagen signature) [6]. Increased radial alignment and direction with respect to tumor cells has been associated with local invasion and transition to metastasis.

Osteopontin is also an important biomarker in cancer [84]. Osteopontin is secreted as a glycophosphoprotein, and then post-translationally modified [91]. Osteopontin is overexpressed in several cancers including breast, lung, skin, and ovarian cancer [91]. It has shown potential as a biomarker for treatment and prognosis in osteosarcoma, as patient survival and therapeutic efficacy in osteosarcoma is correlated with osteopontin overexpression [91]. Similarly, fibronectin has also shown promise as an ECM marker for malignancy [92]. Researchers have developed antibody fragments to help detect fibrin as a tumor marker [92]. Additionally, fibronectin targeting has been used along with MRI to detect micrometastases [93].

### 3.4. In Summary

Given the development of drugs and drug-like molecules that inhibit and generally act on ECM components (through harnessing biology), drug carriers can take this a step further in a few ways. First we can improve delivery of these small molecules, which are subject to rapid wash, to the tumor ECM, by complexing them to nanocarriers. Furthermore, the upregulated ECM components have potential as targets to improve localization to the tumor. In terms of priming the ECM, we can recapitulate some of the approaches described earlier. The next section discusses ways to utilize drug carriers to further the ideas discussed so far.

## 4. ECM Targeting and Drug Delivery

Macromolecular drug carriers have been employed to improve the pharmacokinetics of small molecule drugs. Both passive targeting by the enhanced permeability and retention effect (EPR) and active targeting through use of targeting ligands have been proposed for tumor targeting. A subset of active targeting is targeting the matricellular components. In this section we first describe the advantages and limitations of ECM targeting, followed by examples of targeted ECM components. We then summarize the ECM-based strategies to improve drug delivery for cancer treatment. The strategies include the use of nanocarriers to modulate the ECM to improve drug penetration, to create a drug depot within the ECM, and to directly modulate the ECM. Finally, we include a briefdiscussion of some immunomodulatory factors that are involved in ECM delivery approaches.

### 4.1. ECM Targeting

#### 4.1.1. Advantages of ECM Targeting

Targeting the ECM carries several advantages. The dense stromal barrier as well as the inefficient lymphatic drainage results in a higher interstitial pressure that favors intravasation and limits diffusion [94,95]. There is a limitation in the diffusion of macromolecules to the cell surface, which can result in a gradient of drug localization [94,95]. Passive accumulation via the enhanced permeability and retention (EPR) effect does not affect the migration and diffusion upon extravasation. Furthermore, diffusion can be limited not only by pore size through the matrix but may also be influenced by electrostatic interactions [96]. Thus it is influenced not only by the size of nanocarriers but the surface charge of the carrier. This has been shown through in vitro models which simulate ECM interactions [97]. Targeting the matrix itself can limit the need to traverse this barrier, resulting in more accumulation at the target site. Targeting the ECM and extracellular drug activity may allow for evasion of a common mode of drug resistance: cellular efflux pumps [98].

#### 4.1.2. Limitations and Barriers to ECM Targeting

ECM targeting faces some problems of heterogeneity, a problem associated with tumors in general. It is also important to be cognizant of the site-specificity of different ECM components (i.e., their proximity to blood vessels, gradients within the ECM, etc.). Finally, the ubiquity of ECM processes and targeting sites leads to non-specific localization and action, a problem inherent in most cancer treatments. Targeting ECM components with greater upregulation in the ECM can help to improve tumor selectivity.

#### 4.1.3. ECM Components as Targeting Peptides and as Delivery Targets

Interactions between ECM components and with cell surface receptors have provided information on specific epitopes that are involved in integrin binding [99]. Here we look at some of the matrix components that can serve as delivery targets. Table 3 lists a selection of ECM proteins and targeting peptides known to bind to them. Some examples of matrix targets include collagen, laminin, fibronectin, chondroitin sulfate, tenascin-C, heparan sulfate, and aggrecan [99,100].

##### Fibronectin as a Target

Fibronectin is a glycoprotein that is abundant in the ECM and upregulated in tumors. It regulates a wide range of processes in the ECM. Upregulated fibronectin is an indicator of epithelial-to-mesenchymal transition. Fibronectin has been explored as a binding target, in complex with fibrin [101,102]. The structure of fibronectin contains two domains that are frequently used for targeting: extra domain A (EDA) and extra domain B (EDB), with most methods targeting the latter [102]. Extra domain B is particularly important for tumor angiogenesis. Targeting methods for fibronectin employ either antibodies or peptides [102].

A commonly studied example is CREKA (Cys-Arg-Glu-Lys-Ala), a 5-mer peptide which binds to the fibrin-fibronectin complex and is employed as a targeting moiety [101]. PEGylated liposomes modified to incorporate CREKA, and loaded with doxorubicin, had a higher retention in 4T1 breast tumor inoculated mice, owing to CREKA’s stable binding to fibrinogen [111]. Additionally, an invasion assay demonstrated that binding of CREKA to fibrinogen had an inhibitory effect on cell motility through the invasion chamber [111]. Importantly, CREKA shows negligible binding to fibrin-fibronectin complexes in healthy tissue [93].

##### Collagen as a Target

Collagen has also been explored for ECM targeting. Collagen is the most abundant protein in the human body and in the extracellular matrix, and there are both fibrillar and non-fibrillar (network-forming) types [106]. There are 28 different known subtypes of collagen, with collagen type I being the most prevalent. All collagen types display a signature triple helix structure [106]. There are several collagen binding peptides which are inspired by various ECM motifs, and which bind both to intact and denatured collagen [106]. Some examples are given in Table 3. One well-known example of a collagen binding peptide is the collagen binding domain from the A3 domain of the von Willebrand factor [112]. For example, albumin modified with this collagen binding domain has been utilized as a drug carrier for delivery of doxorubicin in a subcutaneous MC38 colon cancer mouse model, and it showed an increase in accumulation [113].

##### Hyaluronan as a Target

Hyaluronan, a non-sulfated glycosaminoglycan in the ECM, is also a common target, due to the CD-44-hyaluronan interaction’s influence on tumor progression [114,115]. CD-44 is a transmembrane protein, and this interaction plays a role in cell motility [115]. Approximately a quarter of tumors overexpress HA [116]. Reducing hyaluronan reduces CD-44 expression [115,117]. Targeting CD-44 receptors to inhibit HA signaling is a common approach related to hyaluronan, as harnessing the interaction between hyaluronan and the cell surface receptors can modulate tumor growth [114]. Hyaluronan itself has also been employed as a targeting moiety for tumor targeting, and it has been used to modify nanoparticles to improve delivery [114].

##### Tenascin-C as a Target

Tenascin C has been targeted using peptides as well as antibodies [100]. Tenascin-C is a large glycoprotein that is upregulated in tumor ECM, and it plays a supportive role in tumor growth, angiogenesis, and metastasis [100]. Interestingly there is little expression of tenascin in healthy ECM [100]. Using nanoparticle delivery (including liposomes and extracellular release strategies), tenascin-C targeting has been used to deliver drugs towards tumor cells as well as cancer-associated fibroblasts [100].

##### Heparan Sulfate as a Target

Heparan sulfate is a glycosaminoglycan that is expressed in the ECM, cell surface, and basement membrane [118]. It is also significantly upregulated in the tumor ECM and has served as a target [100]. Targeting peptides have been employed to direct nanoparticles (often liposomes) to heparan sulfate for tumor localization [100].

### 4.2. ECM Strategies to Improve Drug Delivery and Modulate Tumor Growth and Invasion

**Figure 2:**
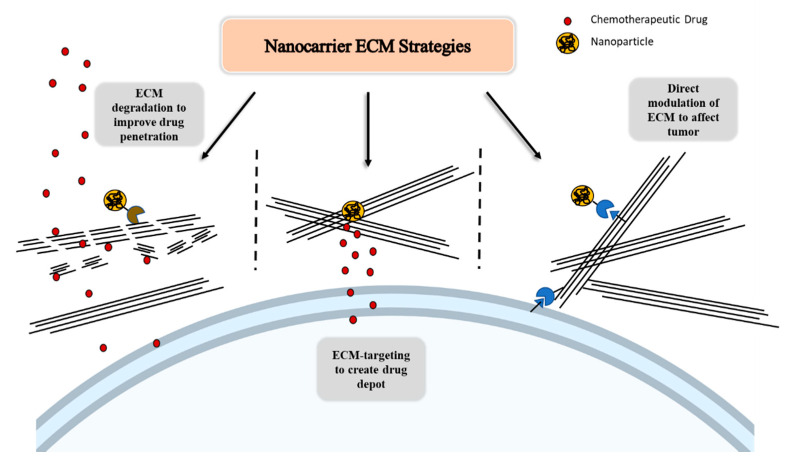
Schematic of extracellular matrix (ECM)-based strategies to improve drug delivery and modulate tumor growth and invasion, with the aid of nanoparticles, through ECM degradation to improve drug delivery, ECM-targeting to create a local drug depot, and direct modulation of the ECM.

ECM-based strategies carry different approaches to facilitate drug delivery, and here we divide them into three broad categories. The first is breaking down the ECM to improve drug penetration. The second is targeting to an epitope within the matrix generally upregulated in order to create a depot for drug delivery to the tumor cells. The third is to target to an epitope in the tumoral ECM to modulate the ECM itself in order to directly impact the tumor. These are depicted schematically in Figure 2.

#### 4.2.1. ECM-Based Strategies to Enhance Drug Penetration through Stroma Modulation

Some cancers are particularly known for their highly desmoplastic stroma, such as pancreatic cancer and some types of breast cancer. Treatment inefficacy is often attributed to an inability to penetrate the ECM. As mentioned earlier, efforts have been made to improve delivery by reducing the ECM barrier. Employing nanocarriers can improve this. One strategy of employing the ECM and nanocarrier targeting to improve drug delivery focuses on reducing the ECM material (which poses a barrier to drug penetration and delivery). Pancreatic ductal adenocarcinoma (PDAC) is often the most utilized application [119]. Many examples of these strategies have found their niche in PDAC, due to the impact of the ECM on its drug exposure. As mentioned earlier PSCs are responsible for ECM deposition in PDAC [120], and the tumor microenvironment is known for its dense desmoplasia and its hypoxic and acidic extracellular environment [121,122]. A greater collagen content in many of these cancers is also correlated with poorer outcomes [123]. Breaking down the stroma also poses the risk of tumor cell escape and metastasis becoming more likely with increased pathways for migration.

Nanoparticles are employed to enhance this strategy through improving the delivery of drugs to tumor ECM with the purpose of downregulating the production of ECM material. This has been done through encapsulating either degradative enzymes (to directly break down ECM) or drug molecules (to downregulate the production of ECM material). These methods further capitalize on ECM biology, by employing an ECM enzyme-responsive aspect to trigger payload release or incorporating binding motifs that bind to upregulated ECM components. For example, MMP-2 responsive peptide-hybrid liposomes (liposome-like particles that incorporate MMP-2 cleavable peptides) designed to encapsulate and release pirfenidone (to down-regulate ECM production) at the site of the pancreatic tumors were able to down-regulate ECM material production and increase penetration of small molecules in a model of PDAC [124]. Gemcitabine was delivered with improved penetration and delivery. Delivery of the ECM-altering drug relied on improved targeting of delivery, which was aided by the nanoparticle. In a similar study, collagenase was encapsulated in liposomes (to afford enzyme protection and encourage localization at the tumor) and delivered similarly to break down the ECM in pancreatic cancer [125]. Following this, paclitaxel micelles were delivered resulting in improved delivery. Interestingly, breaking down the ECM in this study did not seem to increase metastasis [125]. Another study used PEGylated polyethyleneimine-coated gold nanoparticles to deliver all-*trans* retinoic acid (ATRA) [126]. ATRA is used to inhibit pancreatic stellate cells, which reduces deposition of ECM material in pancreatic tumors. The nanosystem is pH-responsive, capitalizing on the slightly acidic tumor extracellular pH, generally cited as being between pH 6–7. This strategy led to the inhibition of PSCs in order to restore homeostasis in the ECM. Following this gemcitabine is delivered, and drug penetration is increased due to a decreased barrier function.

Photothermal agents have also been incorporated into nanoparticles for delivery to the tumor ECM, in order to then break down the ECM for drug delivery. Bioinspired lipoprotein nanoparticles loaded with a photothermal agent that can be triggered by near-infrared light irradiation were delivered to a 4T1 orthotopic mouse breast tumor model [127]. The photothermal agent used was DiOC18 (7) (DiR), and its purpose was to remodel the tumor microenvironment to allow for better penetration of the drug mertansine, also loaded into the particles. The nanoparticle allowed for increased accumulation at the target site, and administration of near infrared light irradiation allowed for the activation of photothermal effects to remodel the ECM in favor of promoting penetration. The improved drug delivery was effective in killing cancer-associated fibroblasts and tumor-associated macrophages. This study represents another important concept because it addresses these supportive cells present in the ECM which contribute to tumor proliferation, strategically combining aspects of the ECM’s contribution to tumors.

These examples demonstrate that the enzymes that break down the ECM can improve delivery using nanocarriers, and this in turn improves small molecule delivery through the ECM for treatment. Furthermore, nanocarriers can deliver other ECM degraders to help prime the ECM.

#### 4.2.2. Utilizing the ECM as an Attachment Site for a Drug Delivery System

Another often employed strategy utilizing the ECM is in targeting sites within the matrix for localization, and then releasing drugs for delivery to the cells. In this fashion nanoparticles targeted to the ECM serve as a localized depot. The value of doing this is to improve penetration into the tumor microenvironment, reducing the need for a drug to diffuse across this barrier. Utilizing the ECM for targeting and as a drug depot is further beneficial compared to targeting cell surface receptors, as intratumoral heterogeneity may result in targeting some cells while leaving others. Furthermore, it will also directly affect cancer-associated cells, which may not display the same upregulations. Lastly an active targeting strategy to the ECM may also see a greater differential in upregulation compared to one targeting cell surface receptors [100].

Researchers have applied this concept to treat epithelial ovarian cancer, using for example the single chain antibody GD3G7, which binds to chondroitin sulfate, incorporated into a lyophilisome. Chondroitin sulfate is highly expressed in ovarian cancer. The lyophilisomes were loaded with doxorubicin. It was validated that the lyophilisome was bound to chondroitin sulfate and the drug load released extracellularly, demonstrating the ability to create a depot within the ECM [128]. Another study similarly used a triple negative breast tumor 3D spheroid model and demonstrated that liposomes targeted to the ECM could serve as a depot releasing cisplatin to alter the distribution of drug molecules within the matrix [129]. On the premise that many ECM components are negatively charged, they used a positively charged nanoparticle to be attracted to the ECM. An in vivo study showed that this nanoparticle reduced growth rate in tumors. A similar study used pH-sensitive liposomes designed to be triggered to release cisplatin load in an acidic environment [130]. They found that cell viability decreased with delivery using these pH-sensitive liposomes, and that the distribution increased. Again targeting the matrix here addressed the purpose of improving drug distribution within the tumor microenvironment.

These examples illustrate the utility of the ECM as a target, some of which are noted in Table 3. They also illustrate pH-mediated extracellular drug release in the mildly acidic tumor ECM. Finally, they illustrate the idea of delivering drugs to cells, but the concept can be extrapolated to non-cellular targets as well, as is exemplified in the next section.

#### 4.2.3. Modulating the ECM to Reduce Tumor Growth and Invasion

Another important strategy involves direct modulation of the ECM. This addresses the important influence that the ECM has on cancer growth and especially invasion and metastasis. As discussed earlier in this review, the ECM contributes significantly to delivering cues to cells, and modulating it can have a direct result on growth and invasion, as separate from priming the ECM for other drug delivery to cells. Modulation of the ECM in conjunction with cytotoxic approaches may prove to have additional advantages. Here we present examples that either directly modulate the ECM or deliver small molecule drugs that modulate the ECM.

PLGA nanoparticles incorporating batimastat (first generation MMP inhibitor) were delivered to treat hepatocellular carcinoma (HCC) [52]. Batimastat inhibits MMPs, in order to directly modulate the ECM remodeling, and this results in slowing of angiogenesis. Treatment was done in conjunction with transarterial chemoembolization (TACE), which is the primary treatment for HCC and often results in stimulating angiogenesis to compensate for the embolized blood vessels. The strategy employed here is that by using batimastat to inhibit MMPs, angiogenesis could be slowed, supporting the TACE treatment. Thus a combination of nanoparticles and ECM modulation is used to aid another treatment hampered by angiogenesis. Nanoparticles can also be used to deliver ECM-modulating drugs. For example, a study demonstrated the use of lysolipid-containing thermosensitive liposomes to deliver marimastat (an MMP inhibitor) to the breast tumor ECM in order to suppress the ECM remodeling which contributes to metastasis, demonstrating suppression of lung metastasis [131].

These studies exemplify the use of nanocarriers to modulate the ECM, as well to play a supportive role in another form of treatment (TACE), which would be a fruitful use of ECM tools. Table 4 provides a summary of ECM-based strategies.

Finally, it is important to note the immunomodulatory factors that may be associated with ECM targeting and modulation. The tumor microenvironment fosters immune suppression through many pathways. For example, collagen deposition is associated with immune modulation, specifically through functioning as a receptor in immune inhibitory signaling [132]. Disruption of these pathways may have unintended consequences in immune suppression. For example, suppression of the CAFs associated with the aforementioned collagen deposition has also been shown to contribute to immune suppression through other pathways [132].

## 5. Discussion and Future Directions

At this time, clinical studies employing ECM-based approaches are very limited. While some examples of ECM-acting drugs have progressed to clinical trials (with many obstacles to their success, particularly delivery), there is not a wealth of information on the translatability of these delivery strategies. Furthermore, the heterogeneity of the tumor microenvironment poses a significant obstacle. Many of the biomarkers and epitopes targeted in these strategies have varying levels of overexpression in different patients and at different stages, underscoring the need for a personalized approach. A key merit of ECM-based approaches is that they move away from oversimplifying the tumor microenvironment, by considering the specific location of different ECM components as well as the limits to migration and diffusion within the site. It also takes into consideration the supportive and responsive nature of the tumor environment, including for example pancreatic stellate cells, which critically limit the efficacy of drugs. The drug delivery field has advanced to incorporate and adapt many of its strategies (polymers, antibody-drug conjugates, inorganic nanoparticles, etc.) to address the ECM. Furthermore, strategies have been appropriately applied to cancers in which the ECM is the most significant barrier, notably pancreatic cancer, ovarian cancer, and breast cancer. The ECM provides ample opportunity as a battlefront in the treatment of cancer, and a solid biological understanding is key to harnessing its potential. Furthermore, an understanding of the local stromal biology can aid even other delivery strategies, which sometimes oversimplify the tumor to a homogenous site immediately presenting itself after drug extravasation. There are many elegant approaches to harnessing the ECM’s contribution to tumor biology to aid in treatment, both direct and indirect methods. This includes priming the ECM, directly modulating the ECM, delivery to matrix targets, therapeutically targeting the ECM, and other combinations which can facilitate treatment, and these methods are further enhanced through the wealth of information on nanocarriers. The ECM’s most important role is in invasion and metastasis, and addressing the ECM to slow metastasis can be done in conjunction with cytotoxic approaches. Nanocarrier approaches can greatly benefit from a knowledge of the ECM’s biology and influence, and similarly ECM approaches can greatly benefit from improvements in carrier technology to improve delivery and targeting.

## Figures and Tables

**Table 1:** Extracellular matrix enzymes.

Enzyme Family	Enzyme	Substrate	Ref
MMPs	MMP-1	Type I and II collagen	[37]
	MMP-2	Gelatin, Type IV collagen	[38]
	MMP-3	E-cadherin, laminin, Type IV collagen; activates cytokines and growth factors; activates MMP-1, -8, -13, -9	[39]
	MMP-7	Type IV collagen, fibronectin, vitronectin, elastin, aggrecan	[40]
	MMP-8	Type I, II, and III collagens, gelatin, aggrecan, fibronectin	[34,41,42]
	MMP-9	Gelatin, Type IV collagen	[38]
Cathepsins	Cat B	Type IV collagen, laminin, fibronectin	[36]
	Cat K	Type I collagen, particularly bone	[35]
	Cat L	Type I and IV collagen, laminin, fibronectin, and elastin	[43]
	Cat S	Collagen, Elastin, E-cadherin	[44]
ADAMs	ADAMTS-18	Chondroitin sulfate	
Other Proteinases	Lysyl oxidase	Crosslinks elastins and collagens through conversion of lysines	[45]

**Table 2:** Inhibitors of extracellular matrix enzymes.

Inhibitor Family	Inhibitor	Type of Inhibitor	Enzyme	Clinical Use/Translation	Ref.
MMP Inhibitors	Batimastat	Small molecule	Broad spectrum	Ended at Phase III	[51,52]
	Marimastat	Small molecule	Broad spectrum	Ended at Phase III	[53]
	Tanomastat	Small molecule	MMP-2, -3, -9	Ended at Phase III	[54]
	Doxycycline	Small molecule	Broad spectrum	Ongoing	[54]
	TIMP-1, -2, -3, -4	Endogenous inhibitor	Broad spectrum	n/a	[55,56]
	SDS3	Antibody	MMP-2, MMP-9	n/a	[55,57,58]
	Prinomastat	Small molecule	Broad spectrum	Ended at Phase III	[59]
Cathepsin Inhibitors	L-235	Small molecule	Cat K	n/a	[60]
	Relacatib	Small molecule	Cat K, B, L, S V	Ended at Phase I	[35,61]
	Odanacatib	Small molecule	Cat K, B, L, S, V	Ended at Phase III	[35,62,63]
	E64	Small molecule	Cat B, Cat L	n/a	[64]

**Table 3:** Extracellular matrix components and associated targeting moieties.

Matrix Targets	Targeting Peptides/Antibodies	Reference
Fibronectin	CREKA	[101]
	CLT1	[101]
	CLT2	[101]
	F8 antibody	[102]
	L19 antibody	[102]
Collagen	CNA35	[103]
	WYRGRL	[104]
	Collagen mimetic peptides	[105]
	TKKTLRT	[106]
	WREPSFMALS	[107]
Tenascin-C	FHKHKSPALSPVGGG	[108]
	F16 antibody	[108]
Hyaluronan	CKRDLSRRC (IP3)	[109]
Heparan Sulfate	NT4	[110]
	CGKRK	[100]

**Table 4:** Strategies Employing the Tumor ECM.

	Types of Nanocarrier Delivery Systems	Examples and Remarks
Targeting ECM Components	Polymer nanoparticles [52]	Matricellular targets include: Collagen [112,113], Fibronectin [101,102]. Laminin, Hyaluronan [114], Tenascin-C [114], Heparan sulfate [100]
	Antibodies [114]	
	Liposomes [114]	
Modulating ECM to reduce barrier to delivery	Liposomes [124]	Breakdown of matrix through direct breakdown or reduction of matrix expression
	Gold nanoparticles [126]	
	Lipoprotein nanoparticles [127]	
Using ECM as a local drug depot	Liposomes [128,129,130]	
	Lyophilosomes [129]	
Modulating ECM to directly alter tumor growth and invasion	Liposomes [131]	MMP inhibitors [131]
	Polymer nanoparticles [52]	
